# How can we enhance ‘real-time’ patient involvement in medical education? A qualitative study of patients and students

**DOI:** 10.1186/s12909-025-06767-x

**Published:** 2025-02-10

**Authors:** Adedoyin Alao, Bryan Burford, Hugh Alberti, Gillian Vance

**Affiliations:** School of Medical Education, Faculty of Medical Sciences, Newcastle University, Newcastle upon Tyne, NE2 4HH UK

**Keywords:** Patient involvement, Medical education, Undergraduate training, Active involvement, Teaching consultations, Medical students, Bedside teaching, Primary care, General practice, Active patient involvement

## Abstract

**Background:**

Patient contact is integral to undergraduate medical training. While political strategies emphasise the ‘patient’s voice’ in medical education, the literature on how to enhance the active involvement of real-time patients is sparse. Increased demands for real-time patient interactions in primary care poses a challenge for educators to provide adequate opportunities for students to learn. Evidence on how students may benefit from patients’ active involvement may inform the design and construction of teaching/learning encounters to optimise the educational value while maintaining positive patient experiences. This study aimed to identify how the involvement of real-time patients in medical education might be enhanced. It examined two research questions: •How does enhanced patient involvement support student learning in different areas of practice? • What can be done to enhance patients’ active involvement in medical students’ training to support those learning benefits?

**Methods:**

We conducted focus groups with patients, workshops and interviews with medical students and analysed the data using codebook thematic analysis.

**Results:**

Patient contact helped students develop their knowledge, clinical and interpersonal skills, professional values, confidence and sense of identity. Students learn by practising the role of a doctor, observing clinicians and reflecting on their experiences. Real-time patients provided experience of diversity, real-life stories and contextual variations that promoted learning. Patients would like to perform active roles, such as shaping encounters and providing explanations, feedback and emotional support to students. While such active involvement may provide learning opportunities for students, many patients were unable to perform active roles during their previous interactions with students. Patients’ active involvement may be facilitated by ensuring adequate introductions, good relationships and an explicit invitation.

**Conclusion:**

The resulting model may form the basis for future research and interventions that encourage and support patients to actively participate in teaching consultations. [293 words]

**Supplementary Information:**

The online version contains supplementary material available at 10.1186/s12909-025-06767-x.

## Background

Patient contact is fundamental to undergraduate medical education, enhancing students’ motivation to learn, clinical reasoning and communication skills and promoting empathy [1–3]. Students learn by participating in practice through observing, rehearsing, or performing the role of clinicians [4], thereby developing competencies and states of mind that are characteristic of a doctor. Students also learn through a cognitive process of reflecting and making meaning of their experiences, thereby informing future practice [5, 6]. Patients also derive benefit from participation in training through the fulfilment of altruistic desire, improved knowledge, and self-esteem [7, 8].

Historically, patients were passively involved in teaching by illustrating symptoms and procedures [9]. However, changes in social and demographic characteristics of the population have given rise to an increased emphasis on patient-centeredness, which is now expected to form the basis of health professional education [10]. The need for active patient involvement has been highlighted in healthcare and educational policies [11–13]. Current GMC guidance states that patients should be invited to become ‘partners in education’, and their role as ‘active teachers’ must be acknowledged [9]. Active patient involvement, where patients engage directly with the students, can improve students’ examination skills [14], confidence, respect for patients and understanding of disease experiences [15]. Conversely, passive involvement, where patients are merely present as objects for students to learn from, may make patients feel embarrassed and resentful [16]. Bleakley and Bligh [17] recommended a radical overhaul of medical education that places the focus of learning on the patient-student relationship, with the doctor playing a supportive role.

While medical education often involves ‘expert’ patients who are recruited and trained specifically to support undergraduate training [18, 19], students also benefit from interacting with patients who attend in real-time with undifferentiated clinical problems and who are opportunistically involved in training. These ‘real-time’ patients create unique learning opportunities by allowing students to encounter patients with diverse clinical problems and backgrounds and more closely represent the practice of newly graduated doctors. However, real-time patient involvement is often passive [20], and active involvement of these patients is complicated by the dual purpose of patient care and teaching. Little is known about the barriers to and/or facilitators of the active involvement of real-time patients. Hence, there is a need to develop a greater understanding of the perceptions of real-time patients about their roles and how these roles may be facilitated.

The increasing number of medical students [21] and the expansion of remote healthcare delivery pose challenges to providing students with adequate opportunities to learn from real-time patients. Primary care placements offer students valuable opportunities to see a wide range and volume of patients and learn about the sociocultural context of illness [22, 23], thereby equipping future doctors to manage complex patient needs. However, few studies have examined students’ views about learning from real-time patients in primary care. Those that have been published have shown that interactions with real-time patients increase students’ motivation, confidence, satisfaction, and sense of identity and may provide the best contexts for developing clinical skills [24–26]. Only one study [24] provided evidence from both patients and students, and this study was conducted over a decade ago.

Our current study aimed to identify how the involvement of real-time patients in medical education might be enhanced, given the increased demands for real-time patient experiences. Insight into how students might benefit from active patient involvement in this context may indicate ways in which teaching/ learning encounters may be designed or constructed to optimise their educational benefits while maintaining positive patient experiences. This study contributes to the literature by exploring the views of two relevant parties in clinical learning encounters - patients and students - thereby providing insight into how these encounters can be adapted to maximise the benefits for both parties. It examines two research questions.

- How does enhanced patient involvement support student learning in different areas of practice?
- What can be done to enhance patients’ active involvement in medical students’ training to support those learning benefits?

## Methods

### Conceptual orientation

This study adopted a constructivist perspective, which acknowledges the existence of multiple realities, which are subjective meanings developed through peoples’ interactions with the world [27]. As patient involvement is concerned with interactions between individuals and the shared world of a teaching consultation, the constructivist perspective provides an appropriate lens to examine this phenomenon. This perspective posits that knowledge is co-constructed through interactions between researchers and participants. Hence, researchers’ backgrounds, experiences and interests influence data collection and interpretation.

### Study context

We collected data from four general practice surgeries spread across the North East of England, which train medical students from Newcastle University. These surgeries serve patient populations of nine to twenty thousand with varying sociodemographic characteristics (Additional file 1).

At Newcastle University, medical students train for five years (or four years in a graduate programme). At the time of this study, they attended general practice for two days per year in the first and second years, respectively, half a day per week in the third year, and for three weeks in the fifth year. Here, students interact with real-time patients by observing the consultation, though they may participate to varying degrees, according to the clinician’s discretion. On occasions, they may consult with patients independently, after which they discuss the case with the clinician, who reviews the patient and provides advice on management.

### Reflexivity statement

The principal researcher, AA, was a general practice (GP) registrar who taught medical students. Her clinical and teaching roles informed her understanding of teaching consultations, which would have influenced the type of data collected and its analysis. Her assumptions were challenged, and her insights deepened through discussion with a diverse research team.

The research team included BB, a medical education researcher with a psychology background, who delivers postgraduate education modules and supervises postgraduate researchers; HA, a GP who leads undergraduate GP education and oversees a team of academic GPs; GV, a paediatrician who leads a team of clinical academics with experience in undergraduate education, including simulation; and SH (see Acknowledgments), a behavioural scientist and health services researcher with expertise in patient and public involvement in research. All team members share an interest and expertise in medical education research, with AA pursuing a Doctorate degree at the time of the study. Team members contributed insights from educational theory and practice that influenced the study’s design. For instance, practical knowledge about curriculum delivery within the region guided decisions about participant recruitment and the structure of discussion schedules.

Additionally, the team held monthly data analysis meetings, where members compared their interpretations of the data. These discussions encouraged AA to critically reflect on her assumptions about the data and develop a more nuanced understanding of the findings.

### Participants and recruitment

Patients who had completed an initial questionnaire survey [28] were invited to participate in a focus group and selected purposively to ensure diverse sociodemographic characteristics, experiences with students and inclination to participate. Adult patients who had attended the surgeries with ongoing clinical problems were eligible to participate in the study.

We invited students from all years of training through social media, emails, and a medical education research student group.

### Data collection

We collected data between June 2016 and November 2017 using focus groups with patients, workshops and interviews with students (Table 1).

**Table 1:** Methods for data collection

Method	Medium	Duration
Focus groups with patients	In-person	90–120 min
Workshops with students	In-person	120 min
Interviews with students	In-person and telephone	49–97 min

In-person focus groups with patients used a semi-structured approach. Two members of the research group moderated the discussions, using a focus group schedule as a guide (Additional file 2) but allowing the discussions to be guided by participants’ responses. We developed the schedule iteratively to explore emergent themes.

In-person workshops with students built on the data from the patient focus groups. We conducted workshops to facilitate dissemination of the patient data and encourage reflection and dialogue among students. We presented the patient data to students to stimulate reflection about how patient views related to their own experiences with patients. We then asked students to recall memorable experiences with patients, and identify what they valued most from the interactions. These activities enabled the students to develop understanding of patients’ perspectives, providing valuable context for the ensuing discussions.

Using a discussion schedule (Additional file 3), we then discussed the issues identified from the patients’ data as well as the students’ perspective of learning. Fewer students than hoped for attended the workshops; hence, we organised interviews with students to supplement the workshop data. The flexibility offered in timing and locations encouraged more students to participate in the interviews.

We developed an interview schedule (Additional file 4) to further explore issues raised in the students’ workshops. AA conducted one-to-one interviews, using a semi-structured approach, either face-to-face or via telephone.

We audio-recorded the qualitative data, with a median duration of 90 min.

### Data analysis

Qualitative data were transcribed, anonymised and analysed using codebook thematic analysis [29]. Data analysis was led by AA, who worked collaboratively with team members through monthly meetings where members shared perspectives on data interpretation. First, AA familiarised herself with the data by reading the transcripts repeatedly and checking them against the audio recordings for accuracy. Next, AA organised the data into codes, which were developed inductively (interpretations were driven by the data) and deductively (with an emphasis on addressing the research questions). These codes were discussed with the research team, who agreed on codes for the first transcript. AA applied these codes to the remaining transcripts and developed further codes as the analysis progressed. These codes were analysed to form themes, and narratives were developed. The research team discussed themes, which were developed iteratively during data analysis and narrative development.

Patient and student data were analysed separately and written up as part of a doctoral thesis. Codes from both datasets were then reanalysed together, and new themes were generated, integrating views from both groups.

### Ethics

Ethical approval was granted by the UK Health Regulation Authority following proportionate ethical review in 2016 (REC 16/NW/0159). Informed consent was obtained from all participants in the study.

## Results

### Participants

Twenty-three patients participated in four focus groups, seven students participated in two workshops, and five students participated in interviews, resulting in a total of thirty-five participants (Table 2).

**Table 2:** Participants’ characteristics

	Male	Female	Gender not specified
Patient focus groups	12	10	1
Students’ workshops	1	6	
Students’ interviews	3	2	
Total	16	18	1

### Themes

We developed themes that describe what students gain from interacting with patients, patients’ roles during these interactions, and how they may be supported to perform active roles.

### Students’ learning gains

Patients were motivated to support students’ training. They felt that their participation could help to develop the healthcare workforce, thereby benefiting society, and that this benefit should be highlighted when patient participation is being promoted.

Both patients and students described how students may benefit from patient contact. These included developing an understanding of disease presentations, clinical and interpersonal skills, professional values, and affective states.

### Understanding of disease presentations

When students encountered patients with a particular illness, they formed a mental picture of these patients with the presenting features, called illness scripts. They used these mental pictures to anchor their knowledge of the illness, which helped them to memorise its characteristics. Students expanded their illness scripts by interacting with patients who presented in various ways. Patient participants stated that the broad and varied exposures afforded by real-time patients enable students to appreciate how disease presentations may differ among patients and adopt a holistic approach.

*‘All patients are individuals*,* they’re not just an illness or a label*,* and the presentation of that illness and its impact on different individuals is very varied and some may have very atypical symptoms*,* rather than stereotype textbook kind of symptoms which can be illustrated in such an encounter.’ (Male*,* 61–75 years)*.

Students valued hearing stories from real-time patients, as these stories were more natural and engaging than those of simulated or expert patients. The rich and personal descriptions helped students remember the features of diseases.

*Patients that are very descriptive*,* like the pain was like giving birth… they make it very personal and that makes it memorable because when I look at my notes*,* I immediately remember exactly how the patient described the symptoms (3rd year male student).*

Listening to patients’ stories also helped students appreciate patients’ beliefs, social contexts of disease, impact of disease on patients, and experiences of healthcare.

### Clinical and interpersonal skills

Patients and students explained that real-time encounters enable students to have hands-on experience practising their skills. Students stated that they could perform intimate examinations as part of their clerking, which they felt uneasy conducting on simulated patients because of the absence of a clinical need. Students valued their experiences in general practice, where they were assigned patients to assess independently. Clerking patients with undifferentiated symptoms challenged students to elicit abnormal signs during examinations, which helped to improve this skill.

Practising with real-time patients provided additional benefits over manikins or simulated patients, as students must adapt their techniques because of contextual variations, such as body habitus, skin colour, and gender, which challenges them to develop their competence. One participant explained that students would be challenged to adapt their technique when inserting an intravenous cannula for her because of her body habitus.

*‘Say well they’re trying for an injection; we play this game called hunt the vein.’ (Gender not specified*,* 46–60 years)*.

Students described the additional challenges, as well as learning opportunities, presented by consideration of patients’ privacy and comfort. For instance, a male student stated that he had not known how to handle a female breast during chest examination. Similarly, while manikins produce regular signs that are easy to elicit, the signs found in patients are more variable and might require them to adjust their technique.

*‘I think there is definitely a real benefit listening to actual patients. Manikins have got very particular sounds; they are normally a lot easier to hear*,* they are meant for you to hear when you put a stethoscope on the chest. Whereas patients*,* they vary in size and weight.’ (5th year male student)*.

Patients stressed the need for students to develop the ability to communicate clearly with patients, establish good relationships, observe, and respond to cues. They felt that these abilities are paramount for good clinical practice to improve patients’ experiences and prevent complaints. Patients felt that students might have limited perspectives of the world because of their socio-economic backgrounds, which might differ from those of patients and affect the way they relate with patients. Thus, they explained that students can develop their interpersonal abilities by interacting with many patients from a wide range of social and cultural backgrounds.

*‘They are only ever going to learn if you let them loose with the great unwashed*,* the public*,* until they can meet them face to face and develop those communication skills*,* learn how to be a doctor in the practical sense.’ (Male patient*,* 46–60 years)*.

Students felt that their interactions with real-time patients who were unfamiliar with clinical procedures challenged them to develop their interpersonal skills, for example, by having to give clear instructions during examinations. They learnt how to respond to cues and manage their dialogue with talkative patients through practice. They also learnt how to structure their histories by observing clinicians.

### Professional values

Students can develop professional values by observing clinicians’ interactions with real-time patients. A few students reflected on clinicians’ practices, which they considered to be negative. For instance, two students disapproved of clinicians’ withholding of information about the severity of illness and limited treatment options from patients. They felt sorry for the patients and thought that the clinicians should have been transparent so that the patients could have realistic expectations.

One student also remarked that GPs sometimes made negative comments about patients’ character, which she felt could be judgemental and may taint students’ opinions of the patients. The pressures associated with real-life consultations may have challenged clinicians’ professional behaviour. Observing how these behaviours could affect a patient made a lasting impression on students to appreciate the value of maintaining good professional attitudes. In addition, the emotions felt by students made these encounters more insightful for students than those with expert or simulated patients who might not trigger such strong emotions.

### Affective learning gains

Students felt that their encounters with real-time patients helped them develop their confidence and sense of identity. Those in the early years of training, or with less experience with certain roles, described feeling anxious about assessing patients or performing procedures. In response, more advanced students reassured them that repeated practice would enhance their competence and thereby mitigate their anxiety. Students developed their sense of identity as doctors by performing legitimate roles within the clinical team, which contributed to their feeling useful and motivated when providing clinical care. This in turn encouraged them to practise and develop practical competencies.

*‘Once you feel like you are actually doing something somewhere you just get so much better at it because you are less frightened to do things wrong.’ (4th year female student)*.

In addition to identifying what benefits students derive from interacting with real-time patients generally, participants also discussed various roles patients can perform in the learning process and how these roles might influence students’ learning.

### Patients’ roles

A few of the patients perceived their role to be as an object for students to practise their skills. However, some felt that they could perform wider roles such as shaping the teaching consultation, teaching, or supporting the students to perform their roles. These patients were keen to perform active roles as they felt that such active involvement could enhance students’ learning from the teaching consultation.

*‘If they want us to critically engage to actually take part in this process from the setting of the agenda right the way through to the review fair enough*,* then that’s where I would like to be.’ (Male patient*,* 46–60 years)*.

Two patients demonstrated that they could shape teaching consultations by identifying learning opportunities. For example, a patient suggested that a student could remove her stitches, which allowed the student to practise the skill for the first time. A few of the patients described teaching roles they had performed, such as asking questions and providing explanations and feedback to the students. They also described providing emotional support to students, particularly when the students appeared anxious, which gave the impression that they needed support. While recalling their experiences, patients expressed their sympathy towards students using phrases such as ‘young man’ and ‘poor thing’. Reflecting on these interactions, patients recognised that students might feel nervous about interacting with patients in clinical settings and could benefit from emotional support provided by patients.

*‘The young man who was in with the asthma nurse was quite anxious. So*,* I asked him a question*,* what he was doing*,* what stage he was at*,* and then he relaxed again into that.’ (Female patient*,* 46–60 years)*.

While only a few of the patients had performed active roles with students, many of them were eager to contribute more but had not had the opportunity to do so. Inability to perform active roles made patients feel dissatisfied as they thought the educational potential of the teaching consultation was not fully harnessed. They felt that patients could demonstrate various ways diseases may present, how they managed their conditions, and provide feedback about students’ skills. Patients and students felt that feedback is a valuable tool for students’ learning, as it is a reliable way of knowing how patients feel about the encounter. Positive feedback can reassure students and boost their confidence, while constructive feedback can help to correct students’ mistakes and develop their skills. Students yearned feedback from patients about their clinical skills as they felt it could help them improve their techniques, for example, ensuring appropriate depth for palpation and position for auscultation. Students also felt that feedback about their manner of interacting with patients could help improve their interpersonal skills. Providing feedback could also be valuable to patients, reinforcing their role in the process.

*‘I just feel the feedback is probably the most important part because you’ve given permission to see a student but then that’s it*,* you’ve been with the student and then nobody knows how you felt about that.’ (Female*,* 61–75 years)*.

However, some patients reported not having the opportunity to provide feedback, and one patient felt her feedback had been ignored when she tried to discuss a negative experience with a GP. She regretted not being able to provide feedback in the same way she would have as a social services manager. This demonstrates that patients can use skills from their personal or professional experiences to actively contribute to students’ learning.

Given the potential value of patients’ active involvement in enhancing patients’ experiences and students’ learning, missed opportunities for such involvement highlight areas for improvement in teaching/ learning encounters.

## Enhancing patients’ active participation

Participants felt that patients may be encouraged to actively participate in training by ensuring adequate introductions, providing an explicit invitation, and establishing good relationships.

### Ensuring adequate introductions and invitations

Reflecting on their own experiences, patients recalled that they had not been explicitly advised on students’ stage of training, level of ability, role, or what might happen during the encounter. Some had guessed the students’ training stage based on the degree of supervision, what happened during the consultation, and the students’ demeanour.

*‘I was on my own*,* he was on his own*,* so I assumed he was in his final year.’ (Female patient*,* 61–75 years)*.

Patients may be reassured by knowing the degree of their supervision. Some students thought that patients might be worried that their consultations would not be reviewed by a clinician. Both patients and students felt that it should be made explicit that the medical student is not yet qualified as a doctor so that patients can set realistic expectations about the student’s performance. Students felt uneasy when their training status had not been clarified during the clinicians’ introductions, as they thought it was deceptive.

A lack of clarity about students’ roles and learning needs might make patients confused about what to expect during teaching consultations, which could make them feel awkward.

*‘I’ve never been in a situation where he’s clarified a role of the person sat there and it’s felt very uncomfortable even though you’ve agreed to it. I’ve found myself trying to encourage the student to get involved in the process not the other way around.’ (Male patient*,* 46–60 years)*.

Patients felt that receptionists should be trained and supported to provide relevant information to patients before teaching consultations. In contrast, students felt that students or clinicians should provide this information. Those in support of students stated that it might encourage them to develop a rapport, while others argued that clinicians can introduce students to patients as a way of letting the students into the patient-clinician relationship.

Patients felt that they needed an explicit invitation to actively participate in a consultation. The absence of this invitation might be a barrier, especially when the clinical tutor is unaware of the patient’s desire or ability to contribute. A patient and a student independently suggested that clinicians should also invite patients to participate in the teaching dialogue with students following a consultation. The patient recalled an experience where the tutor had discussed the clinical case with a student in his absence, which meant that he missed an opportunity to learn about his illness. This made him feel objectified and deprived of the opportunity to benefit from the teaching dialogue.

*‘They were asked to*,* to relay back to the doctor. What the outcome of that was*,* I don’t know. They were just prodding*,* poking*,* asking questions*,* but I was never allowed to hear what their opinion was*,* which might have been beneficial to both of us*,* the student and me.’ (Male*,* 61–75 years)*.

The student added that clinicians may encourage patients to participate in the teaching dialogue by informing patients that they are about to teach, discussing aspects of the presentation that might be easy for patients to contribute to, and using language that the patient understands. This helps to establish a triadic relationship, where patients actively participate in the teaching dialogue between the clinician and the student.

### Establishing relationships

The quality of relationships during teaching consultations affected patients’ experiences and the educational value of the consultation. Students observed that positive relationships made patients feel more relaxed, encouraging them to provide richer accounts of their histories. Additionally, these positive relationships helped students practice their skills, thereby boosting their confidence.

*‘And if a patient is just relaxed… then the student would really have fun and then they will get good at it and then when they are doing it*,* it is like second nature. Whereas if the patient appears nervous then the student is less likely to do it and then the skill isn’t learnt…’ (3rd year male student)*.

Patients and students felt that clinicians play a crucial role in establishing relationships during teaching consultations. When clinicians related well with students, patients were pleased and relaxed. Conversely, poor relationships made a patient feel irrelevant and embarrassed.

*‘You could see that the doctor was bringing them into the conversation*,* which felt very comfortable*,* and I was pleased about that.’ (Female patient*,* 61–75 years)*.

Good eye contact may also help to establish relationships, and this may be achieved by optimising furniture arrangements. Students can also foster relationships with patients by having a friendly attitude and using gestures such as posture, facial expressions, and nodding. These techniques indicate that the student is interested in the patient’s problems and facilitate engagement with the patient.

## Discussion

### Main findings

This study has confirmed the unique value that real-time patient encounters provide to students’ professional development. They bring real-life stories and experiences that are memorable and challenge students to develop their skills. They provide diversity that helps to broaden their understanding of illnesses and social contexts. The emotional aspect of learning about patients’ illnesses was a powerful stimulus for learning. Students developed professional values by reflecting on clinicians’ behaviour and how they might affect patients. Patients wanted to perform active roles to enhance the mutual benefits of the interactions. They needed an invitation, good relationships, and briefing to encourage them to perform these roles.

### Relation to literature

This study supports previous studies showing that patient participation enables students to develop their clinical knowledge and skills [4, 7, 30]. In addition, this study highlights the value of the ‘realness’ of encounters, including the greater degree of challenge, emotional aspects and variability of encounters, which enhance learning. It also describes the mechanisms through which students learn from real-time patients, which include forming illness scripts, reflecting on patients’ stories, and performing legitimate roles.

We found that failure to ensure adequate consent and introductions might make patients feel uncomfortable during the consultation and thereby affect their ability to interact. This finding resonates with previous studies [24, 31], and such practices can make patients feel aggrieved and reluctant to participate in future encounters [31]. Thus, respecting patients’ autonomy may enhance the quality of teaching consultations and encourage future participation.

This study supports previous findings that patients feel disempowered during teaching encounters, which makes them default to passive roles [24, 32], and this may adversely affect their subjective experiences [16]. However, we found that real-time patients are able to actively participate in the teaching process, thereby challenging the idea that their involvement is necessarily passive [10]. Our study extends the literature by demonstrating wider roles they can perform- shaping teaching consultations, providing emotional support, explanations and feedback to students. Such active patient involvement can offer students additional learning opportunities by encouraging them to practice their skills, engage with patients’ stories, and incorporate patients’ feedback into their learning. In addition, promoting the active involvement of real-time patients may enable clinicians to model patient-centredness during clinical learning, helping students appreciate its value in clinical practice.

As suggested by McLachlan et al. [16], this study stresses the crucial role of the clinical tutor in supporting patients’ active involvement by managing relationships during the consultation. Our study also highlights that receptionists and students can promote patients’ active involvement by preparing patients for encounters.

**Fig. 1:**
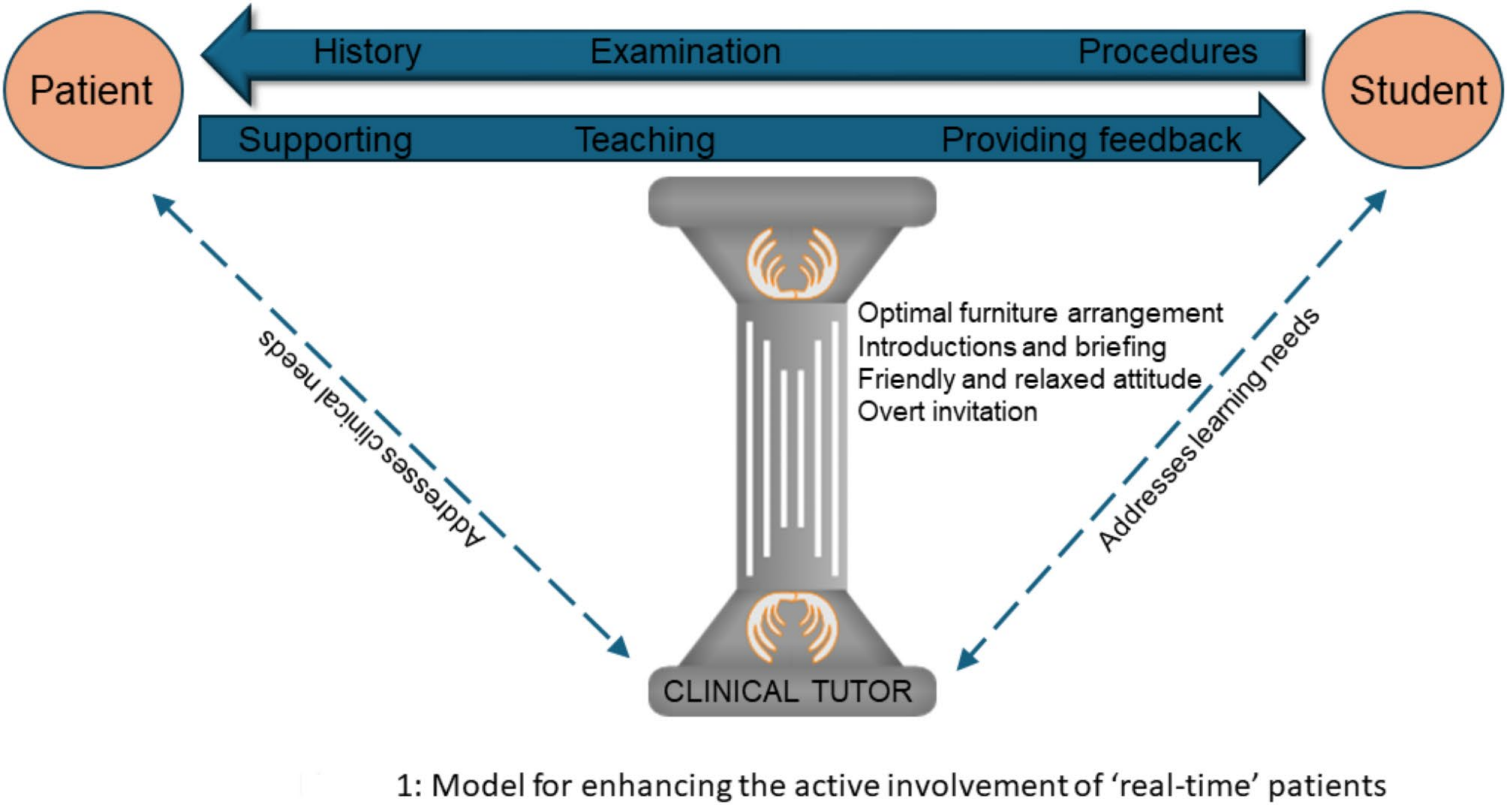
Model for enhancing active patient involvement in medical education

Based on the findings from this study, we developed a model that illustrates how real-time patients’ active involvement may be enhanced (Fig. 1). Teaching consultations involve two activities taking place concurrently, viz., providing clinical care and supporting students’ learning. The clinician is responsible for ensuring that these activities take place optimally, thereby meeting both patients’ and students’ needs.

In addition to allowing students to practise their skills, patients can perform active roles, such as supporting students emotionally, teaching and providing feedback. The clinician may support patients in performing these roles by ensuring appropriate introductions and optimal furniture arrangement, inviting patients to participate in the teaching dialogue, and facilitating relationships. This model provides a greater understanding of the complexities of patient involvement, addressing a research gap identified after a systematic literature review [22]. It may form the basis for interventions to encourage and support real-time patients to actively participate in teaching encounters in clinical settings.

### Practical implications

Our study demonstrates that learning from real-time patients within clinical settings can offer students unique learning opportunities through exposure to diverse clinical presentations and patient populations, role modelling, and reflection on dilemmas and may also support the development of their professional identity. As Dornan et al. [4] warns, reducing students’ learning with real-time patients may have adverse effects on their development as future doctors.

Students develop professional values by reflecting on experiences within the hidden curriculum, which are unintended lessons, values, or perspectives that are learnt within the course [33]. While these students formed positive values by reflecting on negative experiences, others may inadvertently develop negative values after witnessing similar experiences. Clinical tutors may be encouraged to model positive behaviours by supporting them in performing their roles - which may be stressful - and paying attention to their emotional needs [34]. Enlightening them on how their attitudes might influence students’ professional development may also promote positive modelling.

Patients emphasised the value of good interpersonal skills during healthcare provision. They felt that these skills may help to enhance patients’ clinical care and satisfaction and reduce complaints. Students can be supported in developing these skills by interacting with a wide range of patients from diverse backgrounds, an opportunity provided by real-time patients. Thus, encouraging real-time patient participation through emphasising its benefits and improving their experiences of participation may help to prepare students for their future clinical practice.

Our study has revealed that patients want to perform wider roles in students’ learning but often do not have the opportunity to do so. This represents a valuable resource that patients can offer, which is not being effectively utilised. To harness this unique resource, clinicians need to acknowledge the patients’ expertise and support them to actively participate. Developing a framework for promoting active patient participation and enlightening clinical tutors about its value may encourage them to recognise patients’ role as partners in education.

### Implications for further research

Research to test the feasibility of the model developed from this study is required to enhance its applicability. As the model demonstrates that clinicians play a key role in managing teaching consultations, research exploring clinicians’ perspectives on how they might support patients’ active participation is recommended.

### Strengths and limitations

While analysing multiple datasets can be challenging and time-consuming [35], our integration of data from patients and students provides a degree of triangulation, providing robust evidence for interventions that are likely to be acceptable to both parties. The study utilises rich data from various methods - focus groups, workshops, and interviews– thereby providing a more rounded and nuanced understanding of the complexity of patient involvement that would not be possible with a single method [35]. The reflexivity of the research team, which consists of clinical and nonclinical researchers who provided insights into the analysis and interpretation of the data, increases its credibility. A limitation of this research is that there was little representation of younger patients and those from ethnic minorities, which means that the views of these groups are underrepresented. In addition, while clinicians facilitate patient-student interactions in clinical settings, their perspectives on optimising these interactions were not included in this study.

## Conclusion

‘Real-time’ patient encounters provide valuable learning opportunities for medical students. These patients can offer unique learning resources to students by actively participating, and factors affecting this active participation have been identified. This study provides insights that may form the basis of further research and interventions to optimise the benefits of teaching consultations for all.

## Electronic supplementary material

Below is the link to the electronic supplementary material.

Supplementary Material 1

## Data Availability

The datasets generated and/or analysed during the current study are not publicly available to maintain participants’ confidentiality but are available from the corresponding author upon reasonable request.
