# Supplementary material for: How can we enhance ‘real-time’ patient involvement in medical education? A qualitative study of patients and students
Source: BMC Med Educ. 2025 Feb 10;25:216. doi: 10.1186/s12909-025-06767-x (PMC11812220; doi:10.1186/s12909-025-06767-x)
Supplement: Supplementary file 1 — Supplementary Material 1 [file 12909_2025_6767_MOESM1_ESM.docx]

Additional files

Additional file 1 Demographic characteristics of patients within each general practice where patients’ focus groups were conducted

|  | Linthorpe Surgery | Yarm Medical Practice | Skerne Medical Group | Lane Ends Surgery |
| --- | --- | --- | --- | --- |
| Male: Female ratio | 1.043:1 | 0.969:1 | 0.982:1 | 1.053:1 |
| Deprivation Score* | 1^st^ decile | 10^th^ decile | 6^th^ decile | 4^th^ decile |
| Ethnicity | 87.7% White British,  10.5% Non-White ethnic groups,  1.8% Mixed | 96.0% White British,  2.8% Non-White ethnic groups,  1.2% Mixed | 98.3% White British,  1.7% Non-White ethnic groups | 88% White British,  8.6% Non-White ethnic groups,  2.4% Mixed |

*Deprivation score is a relative measure of the level of deprivation within an area. The most deprived areas are ranked within the 1^st^ decile, while the least deprived areas are ranked within the 10^th^ decile (Public Health England, 2020).

Table 1 Demographic characteristics of patients within each general practice where questionnaire survey and patients’ focus groups were conducted

Additional file 2 Patients’ focus group schedule

| Welcome & introductions.  Introduction to the purpose and format of the focus group. Introduce aim of project  Review of focus group information sheet.  Take written informed consent. | |
| --- | --- |
| Knowledge | What do you understand by being involved in teaching medical students?  What do you need or wish to know about the training that medical students have?  How do you get to hear about what training happens in undergraduate medical programmes? |
| Patient role in medical student teaching | Have you ever been asked about the presence of medical students during your consultation?  Has there ever been a medical student present in your consultation either in the surgery or hospital?  Tell us about that.  In what way might this affect your agreement to having a medical student present during your consultation? |
|  | In what ways do you think you might be able to contribute to the student teaching in surgery? |
|  | Do you think it is a good idea for patients to be involved in medical student teaching?  - do you think it would be useful for them? In what ways?  - would it be useful for you? |
|  | How would you like to give feedback to the student, if asked?  How much feedback would you like to give?  What do you consider as useful feedback |
| Student role in patient consultation | What are the important things to you when you come to see the GP? |
|  | Do you think it is a good idea for students to be involved in your consultation?  - in what ways might they be useful?  - would this be useful for you?  If a student is involved in your consultation, what would you wish to know about the student?  What are you prepared to allow students to do when involved in your consultation? |
| Barriers/enablers | Would there be occasions when you would be more or less likely to be involved in medical student teaching in the GP surgery? Tell us about these. |
| Support | Are there things (resources/information) that might be useful to help you participate in medical student teaching?  What might these be? |
| Consent | What were your expectations when asked about the involvement of medical students in your consultation? |

Table 2 Patients’ focus group schedule

Additional file 3 Programme for students’ workshops

| Welcome & introductions.  Take written informed consent.  Reflect on previous experience with patients.  Share data from patient focus groups.  Discuss workshop topics. | |
| --- | --- |
| Feedback from patients | How do you feel about patients giving you feedback? How do you think we can give patient the opportunity or empower them to do so that you can benefit from the feedback?  How do you feel about patients giving you this directly after the encounter? Or giving this to your supervisor? |
| Introduction of students to patients | What are the means by which we can inform patients about the students generally, and specifically, the students they will have an encounter with at a given time?  What do you feel about giving this information to the patients when you see them in the clinical settings? |
| Consent | Based on your experience with patients, what are your views about the way consent is taken?  How do you feel this may be addressed? |
| Practising procedural skills including intimate examinations | How do you feel patients this can be addressed such that patients are comfortable with students carrying out procedures?  How do you know what skills you can practice on patients? |
| Patient/ public education about medical training | What ways do you feel we can make patients more informed about & interested in medical education? What are your thoughts about branding surgeries as teaching surgeries? |
| Feedback to patients about the impact of their involvement | How do you feel we can provide feedback to patients about the impact of their involvement in training? |

Table 3 Discussion schedule for students’ workshop

Additional file 4 Medical students’ interview schedule

| Welcome & introductions.  Introduction to the purpose and format of the interview.  Review of interview information sheet.  Take written informed consent.  Explain the meaning of real-time patient. | |
| --- | --- |
| Explore past experience of student with patient | Tell me about a memorable experience you have had with a real-time patient. This might have been a positive or negative experience.  Has a patient ever refused to see you (or a medical student you know)? Why do you think that was?  What might help to ensure a good interaction between you and the patient? |
| Perceptions about learning from patients | In what ways do patients contribute to your learning? |
| Procedural skills | How do you know what kind of procedure you can carry out on a patient?  How do you judge when you can carry out a procedure on a patient?  What might help to make patients more comfortable with students carrying out procedural skills? |
| Enhancing patient involvement | In what ways do you think patients could be better prepared for participation teaching/learning encounters?  could be encouraged to participate in teaching / learning encounters with students?  Patients tell us they would like to receive feedback about the impact of their contribution to your training. In what ways do you think you could contribute to that? |
| Closing | Is there anything else you would like to ask or say about the involvement of patients in your training? |

Table 4 Students’ interview schedule
